# Repercussions of the COVID-19 pandemic for people with autism and their family members: A scoping review

**DOI:** 10.1590/1518-8345.5965.3729

**Published:** 2023-01-06

**Authors:** Olga Feitosa Braga Teixeira, Samyra Paula Lustoza Xavier, Nuno Damácio de Carvalho Félix, José Wagner Martins da Silva, Rogéria Mônica Seixas Xavier de Abreu, Karla Corrêa Lima Miranda

**Affiliations:** 1 Universidade Federal de Campina Grande, Unidade Acadêmica da Escola Técnica De Saúde de Cajazeiras, Cajazeiras, PB, Brazil.; 2 Universidade Estadual do Ceará, Fortaleza, CE, Brazil.; 3 Universidade Regional do Cariri, Centro de Ciências Biológicas e da Saúde, Iguatu, CE, Brazil.; 4 Universidade Federal do Recôncavo da Bahia, Centro de Ciências da Saúde, Santo Antônio de Jesus, BA, Brazil.

**Keywords:** Autism Spectrum Disorder, Family, Family Relations, Pandemics, Coronavirus Infection, Review, Transtorno do Espectro Autista, Família, Relações Familiares, Pandemia, Infecção por Coronavírus, Revisão, Trastorno del Espectro Autista, Familia, Relaciones Familiares, Pandemias, Infecciones por Coronavirus, Revisión

## Abstract

**Objective:**

to map the diverse scientific evidence available about the repercussions of the COVID-19 pandemic for people with Autism Spectrum Disorder and their family members.

**Method:**

a scoping review conducted in the following databases: MEDLINE, CINAHL, SciELO, SCOPUS, EMBASE and Wiley Online Library, in August and September 2021. The protocol of this review was registered at the Open Science Framework under DOI 10.17605/OSF.IO/JYTVD. The data were analyzed and synthesized in a narrative way.

**Results:**

a total of 46 publications identified indicate that the pandemic period brought about countless repercussions for the population with autism and their family members when experiencing serious difficulties in the changes or irregularities of the scheduled routines and limited access to education, therapies and social support.

**Conclusion:**

the diverse evidence suggests that the COVID-19 pandemic intensified the inequalities already experienced by individuals with autism and their family members, with negative consequences in the emotional, psychological, behavioral and social aspects, interfering with the quality of life and mental health of this population segment. Future studies on individuals with autism and their families during public health crisis periods are of fundamental importance for planning psychiatric, psychosocial and educational interventions.

## Resumo

**Objetivo:**

mapear as evidências científicas disponíveis sobre as repercussões da pandemia da COVID-19 para as pessoas com Transtorno do Espectro Autista e seus familiares.

**Método:**

revisão de escopo realizada nas bases de dados: MEDLINE, CINAHL, SciELO, SCOPUS, EMBASE, *Wiley Online Library*, nos meses de agosto/setembro de 2021. O protocolo desta revisão foi registrado em *Open Science Framework* sob o DOI 10.17605/OSF.IO/JYTVD. Os dados foram analisados e sintetizados de forma narrativa.

**Resultados:**

46 publicações identificadas sinalizam que o período pandêmico trouxe inúmeras repercussões para a população com autismo e seus familiares ao vivenciarem sérias dificuldades nas mudanças ou irregularidades das rotinas programadas; limitação no acesso à educação, às terapias e apoio social.

**Conclusão:**

as evidências sugerem que a pandemia da COVID-19 exacerbou as desigualdades já vivenciadas por pessoas com autismo e seus familiares, com consequências negativas nos aspectos emocionais, psicológicos, comportamentais e sociais, interferindo na qualidade de vida e saúde mental desta população. Estudos futuros sobre indivíduos com autismo e suas famílias durante períodos de crise de saúde coletiva são de fundamental importância para o planejamento de intervenções psiquiátricas, psicossociais e educacionais.

## Resumen

**Objetivo:**

mapear la evidencia científica disponible sobre las repercusiones de la pandemia de COVID-19 para las personas con Trastorno del Espectro Autista y sus familiares.

**Método:**

revisión de alcance realizada en las siguientes bases de datos: MEDLINE, CINAHL, SciELO, SCOPUS, EMBASE, *Wiley Online Library*, en agosto/septiembre de 2021. El protocolo de esta revisión fue registrado en *Open Science Framework* bajo DOI 10.17605/OSF.IO/JYTVD. Los datos fueron analizados y sintetizados en forma narrativa.

**Resultados:**

46 publicaciones identificadas indican que el período de pandemia trajo numerosas repercusiones para la población con autismo y sus familiares al experimentar serias dificultades en los cambios o irregularidades en las rutinas programadas; acceso limitado a educación, terapias y apoyo social.

**Conclusión:**

la evidencia sugiere que la pandemia de COVID-19 agudizó las desigualdades que ya experimentaban las personas con autismo y sus familias, tuvo consecuencias negativas en los aspectos emocionales, psicológicos, comportamentales y sociales, que afectaron la calidad de vida y salud mental de esta población. Es fundamental que se realicen estudios futuros sobre personas con autismo y sus familias durante períodos de crisis de salud pública para planificar intervenciones psiquiátricas, psicosociales y educativas.

**Highlights**

(1) The vulnerabilities of people with ASD were intensified during the pandemic period.

(2) The measures to contain the pandemic caused abrupt changes in everyday life.

(3) These changes favored intensification of psychological and behavioral symptoms.

(4) Quality of life and well-being were impaired in people with ASD and their family members.

(5) The study fills gaps in knowledge and in the national and international literature.

## Introduction

SARS-CoV-2 (COVID-19) is an infectious disease that affects the respiratory system and is caused by the new coronavirus, first identified in December 2019^1^, which, given its propagation ease and the exponential increase in the morbidity and mortality rates, was elevated to the pandemic status in March 2020 by the World Health Organization (WHO)^2^.

Representing the largest global health crisis of the last century, COVID-19 led health authorities in several countries to adopt emergency measures such as mandatory extension of quarantine periods, physical/social distancing, closure of schools and non-essential services, and hygiene and protection procedures with mandatory use of masks, as ways to contain spread of the virus and protect their citizens^3^ ^-^ ^5^.

Although the measures imposed to contain the pandemic are effective in mitigating spread of the virus, they are responsible for multiple changes and disruptions to several aspects of daily life, which have generated unprecedented feelings of tension, fear, stress and anxiety, making the world uncertain, unpredictable and chaotic^5^ ^-^ ^7^.

Among the consequences of COVID-19, there are also the impacts on mental health, as it weakened many individuals’ psychological well-being^4^ ^,^ ^7^, being associated with the onset of psychiatric symptoms in mentally healthy individuals, exacerbation of mental disorders in pre-existing cases and, consequently, increased burden for family members^8^.

The pandemic evidenced and increased social, educational and health disparities^3^, as well as disproportionately affected people with disabilities^9^, including those with Autism Spectrum Disorder (ASD), given the potential for exacerbation of the disorder symptoms and limited access to therapies and social activities, challenging the overwhelming responsibility imposed on their family members^10^.

Based on the foregoing, the relevance of this paper is anchored in the need to direct views and discussions on the COVID-19 repercussions on the health and well-being of people with ASD and their family members, based on the synthesis of diverse scientific evidence, which will favor translation of scientific knowledge into health practice, allowing for the development and implementation of psychiatric, psychosocial, educational and health interventions aimed at this population group. Consequently, the objective was to map the diverse scientific evidence available about the repercussions of the COVID-19 pandemic for people with Autism Spectrum Disorder and their family members.

## Method

### Type of study

This is a scoping review that followed the stages recommended by the Joanna Briggs Institute (JBI) and the Preferred Reporting Items for Systematic Reviews and Meta Analyses Extension for Scoping Reviews (PRISMA-ScR)^11^ checklist, which includes the following: development of the title and question; introduction; inclusion criteria; research strategies; selection of evidence sources; data extraction; analysis of the evidence; and presentation of the results^12^. The protocol of this review was registered at the Open Science Framework under DOI 10.17605/OSF.IO/JYTVD (available from https://osf.io/jytvd).

### Data collection

To formulate the research question, the PPC mnemonic (Population, Concept and Context) was used, where P - People with ASD and their family members, C - Repercussions on the routine/life and C - The COVID-19 pandemic. Consequently, the following was asked: Which is the diverse scientific evidence available about the repercussions of the COVID-19 pandemic on the life of people with ASD and their family members?

The process to search and select the studies took place between August 23^rd^ and September 3^rd^ 2021, in the following databases: Medical Literature Analysis and Retrieval System Online (MEDLINE), Cumulative Index to Nursing and Allied Health Literature (CINAHL), SciELO (Web of Science), SCOPUS, EMBASE and Wiley Online Library (WOL), in three different stages: 1) in the first search, controlled descriptors suitable for the researched databases were used (Medical Subject Headings - MeSH and *Descritores em Ciências da Saúde* - DeCS), connected with the Boolean operators AND and OR; 2) in the second stage, uncontrolled descriptors were used in order to expand the search, using terms specific to the current topic in all the databases chosen; and 3) the last stage consisted in identifying and selecting the sources used from the reference lists. It is noted that it was not possible to include the Gray Literature due to the significant topicality of the theme researched.

The same search strategy was used in all the databases, described as follows: Descriptors (MeSH) - “Autism Spectrum Disorder” OR “Autism Disorder” OR “Autism” AND “Family” OR “Parents” AND “Pandemics” AND “coronavirus disease-19” OR “COVID-19” OR “SARS-CoV-2”.

### Selection criteria

Among the inclusion criteria, all available studies with the most varied methodological designs were selected: comment articles, case studies, editorials, literature reviews, journalistic materials, in all languages and published between January 2020 and August 2021 - when the publications about COVID-19 were introduced in the world literature.

Studies that did not meet the research objectives based on their titles and abstracts were excluded, as well as those unavailable in full after extensive research and copies of duplicate studies, in addition to abstracts from conferences, lectures and/or presentation of papers.

### Data treatment and analysis

Selection of the studies was conducted in a number of phases. In the first, two researchers independently examined the titles and abstracts of potentially relevant studies, and the articles selected that appeared to meet the inclusion criteria underwent a second selection stage. In this stage, the same reviewers independently read the full texts of all the articles selected and excluded those that did not meet the already established criteria. In the case of any divergence during the process to select the articles in the first or second phase, a third reviewer was consulted.

The methodological quality of the primary studies was not evaluated, as this aspect is not considered in scoping reviews. The form recommended by the JBI was used in data extraction in order to ease the information synthesis and the quality of the recommendations^11^.

For mapping the diverse information, data collection took place through an instrument adapted from the JBI form, prepared by the researchers themselves in Microsoft Excel^®^ to record the characteristics of the studies included and the relevant information for the research: publication data (year, authors and country of publication), study objective, methodological characteristics (type of study, characteristics of the population), and main results (outcomes and main findings or contributions).

It is important to emphasize that all forms of data were analyzed qualitatively based on the convergences and/or divergences identified, enabling integration of the ideas in a more summarized and reliable way to the findings.

### Ethical aspects

As this research uses data in the public domain, it waives approval by a Research Ethics Committee; however, all the authorships were duly registered.

## Results

**Figure 1:**
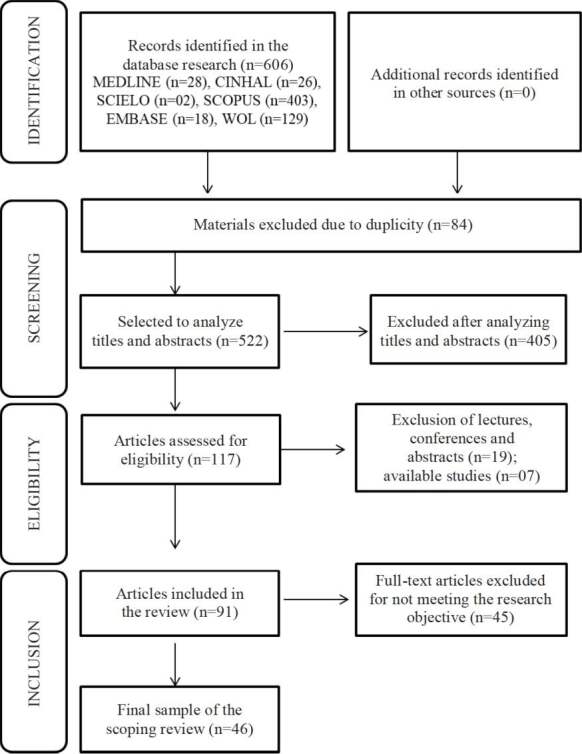
Flowchart of the process to select the studies, adapted from the Preferred Reporting Items for Systematic Review and Meta-Analyses Extension for Scoping Reviews (PRISMA-ScR)^12^. Fortaleza, CE, Brazil, 2021

According to the electronic search, a total of 606 potentially eligible studies were identified in the databases; 84 materials were removed due to duplicity and, after applying the exclusion criteria, 46 articles were read and analyzed by the authors of the study, thus comprising the final sample of the review, as shown in Figure 1.

In order to ease presentation of the data extracted from the articles, Figure 2 includes the characterization of the studies, with information such as title, country, language and methodological approach.

**Figure 2:** Characterization of the articles included in the scoping review. Fortaleza, CE, Brazil, 2021

Citation	Title	Country/ Language	Approach
(3)	*Factors affecting the behavior of children with ASD during the first outbreak of the COVID-19 pandemic.*	Chile/ English	Quantitative
(4)	*Attitude, anxiety and perceived mental health care needs among parents of children with Autism Spectrum Disorder (ASD) in Saudi Arabia during COVID-19 pandemic.*	Saudi Arabia/ English	Quantitative
(5)	*Psychological Impact of COVID-19 Outbreak on Families of Children with Autism Spectrum Disorder and Typically Developing Peers: An Online Survey.*	Italy/ English	Quantitative
(6)	*COVID-19 pandemic effects in people with Autism Spectrum Disorder and their caregivers: Evaluation of social distancing and lockdown impact on mental health and general status.*	Spain/ English	Quantitative
(7)	*The impact of COVID-19 on stress, anxiety, and coping in youth with and without autism and their parents.*	USA/ English	Quantitative
(8)	*The Resilience of Social Service Providers and Families of Children with Autism or Development Delays During the COVID-19 Pandemic - A Community Case Study in Hong Kong.*	China/ English	Qualitative
(9)	*Core experiences of parents of children with autism during the COVID-19 pandemic lockdown.*	Israel/ English	Qualitative
(10)	*The impact of the COVID-19 pandemic on children with autism spectrum disorders.*	USA/ English	Brief Communication
(13)	*COVID-19 and behaviors in children with autism spectrum disorder: Disparities by income and food security status.*	USA/ English	Quantitative
(14)	*Psychiatric problems during the COVID-19 pandemic in children with autism spectrum disorder.*	USA/ English	Quantitative
(15)	*It took a pandemic: Perspectives on impact, stress, and telehealth from caregivers of people with autism.*	USA/ English	Mixed
(16)	*Perceptions of Families of Individuals with Autism Spectrum Disorder during the COVID19 Crisis.*	USA/ English	Mixed
(17)	*Autism and Access to Care During the COVID-19 Crisis.*	USA/ English	Case study
(18)	*Early Pandemic Experiences of Autistic Adults: Predictors of Psychological Distress.*	USA/ English	Quantitative
(19)	*Conducting CBT for Anxiety in Children with Autism Spectrum Disorder During COVID-19 Pandemic.*	USA/ English	Quantitative
(20)	*COVID-19: overcoming the challenges faced by individuals with autism and their families.*	USA/ English	Comment
(21)	*Ten weeks in: COVID-19-related distress in adults with autism spectrum disorder.*	USA/ English	Quantitative
(22)	*Pandemic and Impact on Patients with Autism Spectrum Disorder.*	USA/ English	Comment
(23)	*Brief Report: Impact of COVID-19 on Individuals with ASD and Their Caregivers: A Perspective from the SPARK Cohort.*	USA/ English	Quantitative
(24)	*Ways to support autism & special needs families during the coronavirus pandemic.*	USA/ English	Comment
(25)	*Psychological distress among caregivers raising a child with autism spectrum disorder during the COVID-19 pandemic.*	USA/ English	Quantitative
(26)	*Mental Health and Resilient Coping in Caregivers of Autistic Individuals during the COVID-19 Pandemic: Findings from the Families Facing COVID Study.*	Canada/ English	Quantitative
(27)	*Caregiver burnout, gaps in care, and COVID-19 effects on families of youth with autism and intellectual disability.*	Canada/ English	Case study
(28)	*Coping, fostering resilience, and driving care innovation for autistic people and their families during the COVID-19 pandemic and beyond.*	Canada/ English	Comment
(29)	*Coparenting autistic children during COVID-19: Emerging insights from practice.*	Canada/ English	Journalistic material
(30)	*Supporting children with autism spectrum disorder in the face of the COVID-19 pandemic.*	Canada/ English	Letter to the editor
(31)	*How have youth with Autism Spectrum Disorder managed quarantine derived from COVID-19 pandemic? An approach to families perspectives.*	Spain/ English	Mixed
(32)	*Differences in emotional state and autistic symptoms before and during confinement due to the COVID-19 pandemic.*	Spain/ English	Quantitative
(33)	*The relationship between 2019-nCoV and psychological distress among parents of children with autism spectrum disorder.*	China/ English	Quantitative
(34)	*Impact of the COVID-19 Pandemic on Children with ASD and Their Families: An Online Survey in China.*	China/ English	Quantitative
(35)	*Parental Views of Families of Children with Autism Spectrum Disorder and Developmental Disorders During the COVID-19 Pandemic.*	Turkey/ English	Mixed
(36)	*Behavioral Implications of the COVID-19 Process for Autism Spectrum Disorder, and Individuals*’ *Comprehension of and Reactions to the Pandemic Conditions.*	Turkey/ English	Quantitative
(37)	*Your country is your routine: the evacuation, quarantine, and management of behavioral problems of a child with autism during COVID-19 pandemic.*	Turkey/ English	Case study
(38)	*The psychological impact of the COVID-19 pandemic on adults with autism: a survey study across three countries.*	United Kingdom/ English	Mixed
(39)	*The impact of the COVID-19 pandemic on autistic adults* - *a survey.*	United Kingdom/ English	Mixed
(40)	*COVID-19 and autism: Uncertainty, distress and feeling forgotten.*	United Kingdom/ English	Letter to the editor
(41)	*Stress and emotional wellbeing of parents due to change in routine for children with Autism Spectrum Disorder (ASD) at home during COVID-19 pandemic in Saudi Arabia.*	Saudi Arabia/ English	Quantitative
(42)	*Psychosocial and Behavioral Impact of COVID-19 in Autism Spectrum Disorder: An Online Parent Survey.*	Italy/ English	Quantitative
(43)	*COVID-19 and Mental Health of People with Autism Spectrum Disorder and Their Families; What Can Be Done?*	Iran/ English	Letter to the editor
(44)	*The COVID-19 outbreak and the problems of children with autism.*	Iran/ English	Letter to the editor
(45)	*The impact of COVID-19 on children with autism spectrum disorder.*	Portugal/ English	Quantitative
(46)	*Vécus de familles d*’*enfants autistes en période de confinement: étude exploratoire.*	France/ French	Qualitative
(47)	*Desafios cotidianos e possibilidades de cuidado com crianças e adolescentes com Transtorno do Espectro Autista (TEA) frente à COVID-19.*	Brazil/ Portuguese	Reflective trial
(48)	*Repercusión psicológica en niños con Trastorno del espectro autista durante el confinamiento por COVID-19.*	Cuba/ Spanish	Bibliographic review
(49)	*A study on impact of Corona Virus Disease 2019 Pandemic on activities of daily living, play, and sensory behaviors of children with autism spectrum disorder: A cross-sectional survey study.*	India/ English	Mixed
(50)	*An Unexpected Positive Effect of Social Distancing Measures on the Care of Children with Autism in Vietnam.*	Vietnam/ English	Letter to the editor

Based on Figure 2, it can be seen that most of the publications were in English, had a quantitative approach and were published in 2021, suggesting scientific internationalization about dissemination of the knowledge related to the repercussions of the COVID-19 pandemic in the ASD context.

Regarding the content discussed in the manuscripts, there was standardization in the diverse information published, which was analyzed and organized into categories by content similarity, as follows: 1 - Repercussions of the pandemic for people with ASD; 2 - Repercussions of the pandemic for the family members; and 3 - Benefits of the pandemic for people with ASD and their family members.

Considering the importance of the diverse evidence found and its relationship with the problem question and the research objective, the main results of the studies were organized in Figure 3.

**Figure 3:** Repercussions of the COVID-19 pandemic for people with ASD and their family members. Fortaleza, CE, Brazil, 2021

Repercussions of the COVID-19 pandemic for people with ASD and their family members		
**Repercussions of the pandemic for people with ASD**		
Emotional aspects of people with ASD: - change in general mood^9^ ^,^ ^14^ ^,^ ^17^ ^,^ ^24^; - acute stress^8^ ^,^ ^13^ ^,^ ^15^ ^,^ ^45^ ^,^ ^48^; - increase in anxiety^9^ ^,^ ^17^ ^,^ ^34^ ^-^ ^35^ ^,^ ^48^; - depression^13^ ^,^ ^38^ ^,^ ^42^ ^,^ ^50^; - sleep problems^4^ ^,^ ^26^.	Behavioral aspects of people with ASD: - increase in irritability and/or aggression symptoms^1^ ^,^ ^9^ ^,^ ^17^ ^,^ ^35^ ^,^ ^48^; - inattention/distraction^6^ ^,^ ^35^ ^,^ ^48^; - problematic and self-harming behaviors^14^ ^-^ ^15^ ^,^ ^19^ ^,^ ^33^ ^,^ ^48^.	Changes in the activities of daily living - changes or irregularities in the scheduled routines^7^ ^,^ ^15^ ^-^ ^16^ ^,^ ^49^; - school closures^4^ ^,^ ^15^ ^,^ ^27^ ^,^ ^42^; - difficulty accessing the health services^5^ ^,^ ^9^ ^,^ ^16^ ^,^ ^35^ ^,^ ^47^; - interruption of physical and leisure activities^28^ ^,^ ^31^ ^,^ ^41^; - incipient availability of the social support network^7^ ^,^ ^15^ ^-^ ^16^ ^,^ ^49^.
**Repercussions of the pandemic for the family members**		
- parents’ mental distress^7^ ^,^ ^9^ ^,^ ^31^ ^,^ ^43^ ^-^ ^44^; - incipient availability of the social support network^7^ ^,^ ^15^ ^-^ ^16^ ^,^ ^49^; - professional uncertainties and financial issues^7^ ^,^ ^28^ ^,^ ^42^ ^,^ ^44^ ^,^ ^31^ ^-^ ^32^; - high physical, psychological and emotional burden^8^ ^,^ ^13^ ^,^ ^31^ ^,^ ^41^; - low quality of life^7^ ^,^ ^27^ ^,^ ^31^ ^,^ ^38^; - impaired family dynamics^5^ ^,^ ^7^ ^,^ ^31^ ^,^ ^41^ ^,^ ^44^.		
**Benefits of the pandemic for people with ASD and their family members**		
- improvements in the relationship with their children and family members^4^; - more time to teach new skills related to their autonomy, to establish care routines, and to promote social and communicative interaction skills in their children^7^; - more time for the family and greater family interaction^7^; - the reduction of sensory and social burden during isolation improved their children’s life^4^ ^,^ ^43^ ^,^ ^50^.		

## Discussion

The results referred to in this scoping review made it possible to present a mapping of the production of knowledge about the repercussions of the COVID-19 pandemic for people with ASD and their family members, organized by content similarity in the following categories: Repercussions of the pandemic for people with ASD; Repercussions of the pandemic for the family members; and Benefits of the pandemic for people with ASD and their family members.

The studies indicate that the pandemic period has brought about countless repercussions for the population with autism and their family members, as they experience serious difficulties in the changes or irregularities of the scheduled routines^1^ ^,^ ^13^ ^,^ ^26^ ^,^ ^33^ ^,^ ^38^ ^,^ ^42^, limited access to education and therapies^4^ ^-^ ^5^ ^,^ ^9^ ^,^ ^14^ ^,^ ^33^ and social support^7^ ^,^ ^26^ ^,^ ^43^ ^-^ ^44^, interfering in their quality of life and mental health.

### Repercussions of the pandemic for people with ASD

The findings reveal that the pandemic period brought about negative consequences for the population with ASD in terms of emotional aspects^4^ ^,^ ^8^ ^-^ ^9^ ^,^ ^13^ ^-^ ^15^ ^,^ ^17^ ^,^ ^24^ ^,^ ^26^ ^,^ ^34^ ^-^ ^35^ ^,^ ^38^ ^,^ ^42^ ^,^ ^45^ ^,^ ^48^ ^,^ ^50^, behavioral aspects^1^ ^,^ ^6^ ^,^ ^9^ ^,^ ^14^ ^-^ ^15^ ^,^ ^17^ ^,^ ^19^ ^,^ ^33^ ^,^ ^35^ ^,^ ^48^ and in the activities of daily living^4^ ^-^ ^5^ ^,^ ^7^ ^,^ ^9^ ^,^ ^15^ ^-^ ^16^ ^,^ ^27^ ^-^ ^28^ ^,^ ^31^ ^,^ ^35^ ^,^ ^41^ ^-^ ^42^ ^,^ ^47^ ^,^ ^49^.

The emotional repercussions identified in the population with ASD during the pandemic period^7^ ^,^ ^27^ ^,^ ^45^ ^,^ ^48^ range from milder disorders, such as change in general mood^9^ ^,^ ^14^ ^,^ ^17^ ^,^ ^24^ and acute stress^8^ ^,^ ^13^ ^,^ ^15^ ^,^ ^45^ ^,^ ^48^, to more serious cases, such as increased irritability and/or aggression^1^ ^,^ ^9^ ^,^ ^33^ ^,^ ^42^ ^,^ ^48^, anxiety^9^ ^,^ ^33^ ^,^ ^42^ ^,^ ^48^ ^,^ ^50^, depression^13^ ^,^ ^38^ ^,^ ^42^ ^,^ ^50^, inattention and/or distraction^6^ ^,^ ^33^ ^,^ ^48^, and sleep problems^4^ ^,^ ^26^.

In response to the disruptions imposed by the pandemic, people with autism experienced heightened irritability levels, verbal outbursts and oppositional behavior. Some of them presented a significant deterioration of their behavioral problems, such as hyperactivity, restlessness, decreased adaptation, and impatience. The parents noticed deterioration in communication; increase in stereotyped behaviors, hypersensitivity and aggression; changes in appetite; and emergence of new tics and/or increase in existing ones, as well as self-injury^15^ ^,^ ^26^ ^,^ ^33^ ^,^ ^36^ ^,^ ^40^ ^-^ ^41^ ^,^ ^46^.

The abrupt changes in the routine and the interruption of social activities, therapies and social interaction caused confusion and emotional disorganization among individuals with ASD, due to their preference for highly predictable environments. These circumstances manifest themselves as an involution in the social and emotional behaviors of these patients^26^ ^,^ ^42^.

Closure of schools and discontinuation of the therapies can have a devastating consequence on development of these individuals^42^, as the “break” in the routine represents a factor that generates emotional stress^29^. The transition from face-to-face activities to online education was described as a major challenge for parents and people with ASD alike, in addition to the fact that the online classes further reduced social interaction, triggering feelings of loneliness^14^.

The difficulty accessing the health services was already a reality experienced by individuals with ASD in non-pandemic conditions. The difficulty finding accessible care with overload of the health system, often with additional procedures and restrictions due to COVID-19, intensified the already existing disparities^35^. Inaccessibility to rehabilitation services such as outpatient, speech and occupational therapy can lead to delays in the developmental skills^5^.

Interruption or suspension of the mental health services during the pandemic came at a time when the demand for such services increased exponentially. The difficulty accessing emergency mental health services during the isolation period, the stigma experienced by those suffering from mental disorders and the lack of training in the clinical emergency services to assist this population segment favor the emergence of an epidemic of mental disorders, either concomitant or subsequent to COVID-19.

Isolation forced the interruption of physical and leisure activities for the general population, and had severe consequences for people with autism, as they have a calming and regulating effect, providing stress and anxiety reduction and mood control, and may be linked to the expression of more positive emotions^28^ ^,^ ^31^ ^,^ ^41^.

The incipient availability of the social support network during the pandemic exerted a negative effect both on people with ASD and on the parents, as it is associated with increased caregivers’ frustration due to the work and responsibility overload, as well as to problematic behaviors in the individuals with autism, causing family mental distress^7^ ^,^ ^9^ ^,^ ^31^ ^,^ ^43^ ^-^ ^44^ and negatively interfering with quality of life and family relationships^5^ ^,^ ^7^ ^,^ ^31^ ^,^ ^41^ ^,^ ^44^.

Knowing and considering the changes in the lives of people with ASD during the pandemic allows for a careful analysis of the functioning context regarding the education, health and social assistance services, providing improvements in life and health and mitigating negative effects and sequelae in the development of this population segment in times of public health crises.

### Repercussions of the pandemic for the family members

Caring for a person with ASD is associated with greater parental stress when compared to any other type of disability^32^, and dealing with the pandemic and the restrictive measures is associated with additional demands for these parents.

The COVID-19 pandemic had serious repercussions for the family members of people with ASD, as the literature reports higher stress levels and low quality of life since, in addition to dealing with family and work commitments, they need to carry out complex care activities, such as managing their children’s behaviors and emotions, which are frequently unpredictable^41^. The high level of parental stress can exert a negative impact on the psychological well-being of people with ASD and exacerbate behavioral symptoms, creating a vicious circle^33^.

The magnitude and intensity of COVID-19 presents itself as a serious challenge for parents. Almost all the studies indicated that the economic crisis, professional uncertainties and financial issues^7^ ^,^ ^28^ ^,^ ^31^ ^-^ ^32^ ^,^ ^42^ ^,^ ^44^ resulting from the pandemic generated tensions, anxieties and concerns.

The accumulation of tasks and the need to reconcile multiple functions (home office work, house chores, home education, demands to take care of other family members) during the pandemic, imposed a high physical, psychological and emotional burden on the parents of people with ASD^8^ ^,^ ^13^ ^,^ ^31^ ^,^ ^41^.

All the aforementioned aspects show the vulnerability of this population group. The literature shows that the association of these factors interfered with the parents’ mental health in this pandemic period, and they presented accentuated symptoms of stress^7^ ^-^ ^8^ ^,^ ^27^, anguish^8^ ^,^ ^27^ ^,^ ^41^, anxiety^6^ ^,^ ^8^ ^,^ ^31^ ^,^ ^33^ ^,^ ^44^ and depression^31^ ^,^ ^33^ ^,^ ^44^, as well as poor quality of life^7^ ^,^ ^27^ ^,^ ^31^ ^,^ ^38^.

The families’ mental health has been affected by COVID-19 and behavioral challenges in all family members have been observed. The several interruptions in the daily routine triggered symptoms of anxiety, feelings of tension and concern, compromising family dynamics^29^.

### Benefits of the pandemic for people with ASD and their family members

The people with ASD and their family members experienced the pandemic period differently: some of them found it a very difficult and frustrating time. However, it can be asserted that people were able to readapt and experienced improvements in the relationships with their children and family members^4^.

Among the positive effects of the pandemic found in some literature materials, it is worth noting the fact that some parents stated having benefited from the additional time to teach new skills related to their autonomy, to establish care routines and, perhaps more importantly, to promote social and communicative interaction skills in their children. The isolation period allowed more time for the family and greater family interaction^7^.

In some studies, it was observed that the decrease in the academic demand and social interactions experienced by some children was reported by the parents as associated with a reduction in negative behaviors, improved mood and more displays of affection. The lockdown reduced sensory and social burden in some adults with autism who revealed that the pandemic improved their lives^4^ ^,^ ^43^ ^,^ ^50^.

In general, each family faced the COVID-19 pandemic period in a very particular way; and the socioeconomic situation, the parents’ professional challenges and resilience, access to essential services and the children’s behavior during the quarantine emerged as some of the factors that could/may enhance or minimize the repercussions of the pandemic on the lives of people with ASD and their family members.

Knowledge of how people with ASD and their family members coped with the COVID-19 pandemic is crucial to providing personalized interventions and tailored support in an uncertain period and in the next similar situations arising either from this or from future pandemics^31^.

Discussions about ASD during the pandemic period are relevant, due to the impact on the way in which an individual will feel and interact with the world around them. Despite being a quite delicate aspect for children and also for their parents, through the information and support of specialized professionals it is possible to overcome the challenges and positively deal with this condition that affects millions of people.

### Study limitations

This study was limited by the nonexistence of research studies with a high level of evidence, such as clinical trials and studies with large samples, justified by being a recent theme.

## Conclusion

This paper mapped the scientific production on the repercussions of the COVID-19 pandemic for people with ASD and their family members.

The COVID-19 pandemic quickly generated an unstable situation, which intensified the autistic symptoms and emotional problems and led to drastic changes in the activities of daily living. The prolonged period of social distancing and isolation disturbed the lives of people with ASD and was an important challenge for their family members, interfering with the quality of life, well-being and mental health of individuals with ASD and their family members.

This study is expected to strengthen discussions about how people with ASD and their family members experienced this profound public health crisis and the repercussions it imposed on their lives, in order to drive the production and dissemination of new knowledge in different contexts, in order to achieve improvements in quality of life and to give voice and visibility to this population segment, which lives on the margins of society.

Health interventions and public policies need to be planned and coordinated across all sectors, addressing the range of lockdown-related disparities that people with ASD have experienced during the COVID-19 pandemic and will certainly experience in the future, in order to provide inclusive and responses and minimize harms to this population group in times of public health emergencies.
